# Measuring the Quality of Early Childhood Education in Low- and Middle-Income Countries

**DOI:** 10.3389/fpsyg.2021.774740

**Published:** 2021-10-28

**Authors:** Si Chen, Sharon Wolf

**Affiliations:** ^1^Harvard Graduate School of Education, Cambridge, MA, United States; ^2^Graduate School of Education, University of Pennsylvania, Philadelphia, PA, United States

**Keywords:** early childhood education, low- and middle-income countries, psychometrics, process quality, measurement

## Abstract

Young children’s access to early childhood education (ECE) is increasing in low- and middle-income countries (LMICs), though often without attention to service quality. Monitoring quality requires classroom observations, but most observation tools available were developed in high-income western countries. In this article, we examine key issues in measuring ECE quality in LMICs and consider challenges and opportunities in balancing theoretical grounding, cultural- and contextual-adaptation, and empirical rigor. We then review the literature on observed classroom quality in LMICs, focusing on process quality. We find limited evidence that the constructs identified in high-income countries replicate in LMICs. Further, the very limited evidence that ECE quality measures used in LMICs predict child outcomes is almost exclusively cross-sectional and associations are mixed. We conclude by discussing how future research can build a stronger knowledge base about ECE quality and child development globally.

## Introduction

Access to early childhood education (ECE) in low- and middle-income countries (LMICs) is increasing, yet attention to the quality of these services is only relatively recent (Yoshikawa et al., 2018). Classroom observation tools are part of monitoring the quality of ECE settings. There is a large literature on the conceptualization and measurement of ECE quality in the United States. Efforts to expand access to high-quality ECE in LMICs will require similar efforts that are theoretically grounded and locally adapted. Whether there are universal constructs of ECE quality and the extent that quality is contextually specific is unknown. In this article, we suggest three priority concepts for measuring ECE quality in LMICs and discuss challenges and opportunities for each. These include theoretical grounding, contextual adaptations, and empirical rigor. We then review the nascent literature on measuring ECE quality in LMICs. Finally, we discuss how future research can incorporate each of these elements to build a stronger knowledge base about ECE quality and child development globally.

## Priority Concepts

### Theoretically Grounded

Current conceptualizations of ECE quality are guided by socioecological, constructivist, attachment, and learning theories, which all focus on children’s experiences in their classroom environments and point to the critical role of teachers. *Process quality* broadly describes the nature of children’s daily interactions and experiences in the classroom, including academic, social, emotional, and physical aspects of activities and interactions (Pianta et al., 2005). Attachment theory focuses on the importance of consistent and sensitive interactions with teachers (Ainsworth, 1989); constructivist learning theories focus on the development of cognitive skills through engaging in age-appropriate activities (Gopnik et al., 1999); and Vygotsky’s sociocultural theory focuses on how skilled partners can guide and scaffold children’s learning of more complex concepts (Kozulin, 1998).

Sociocultural theory also highlights the ways that societal, cultural, and demographic factors shape social interactions and learning opportunities (Rogoff, 2003), yet documentation of children’s experiences in ECE settings across diverse contexts is scarce. Whether constructs of quality as currently conceptualized are applicable to, sufficient for, and responsive to the needs of classrooms in LMICs is not well studied (see Tobin et al., 1991 for an exception). For example, while in many high-income western countries, child autonomy is promoted early, in many LMICs there is instead an emphasis on obedience and social responsibility (e.g., Serpell, 2011). Are distinct elements of teacher-child interactions (e.g., classroom management) more predictive of child learning in very large classes, or in cultures where teacher authority is valued? Are markers of quality the same as in high-income countries (HICs) but manifest differently, or are there fundamental differences in the domains of effective teaching across contexts? A deeper theoretical integration of the sociocultural and demographic contexts is needed in global research on ECE classroom quality.

### Locally Adapted

The need for ECE quality measures to meet the realities of the local classrooms in which they are implemented raises some tensions with a universal theoretical grounding. Most existing classroom observation tools are not designed with such breadth in mind. When faced with the selection and use of quality assessment tools, LMIC researchers often encounter the challenges that some of the definitions of “high-quality” do not reflect local cultural contexts.

Current conceptualizations of “quality” in ECE are defined through the lens of HIC contexts and require significant resources to implement, including personalized learning, one-to-one guidance, small group interactions, and dialogic language models (Dahlberg et al., 1999). While it is challenging to implement personalized learning with large class sizes (Gupta, 2004), teachers can create valuable learning experiences through rich and engaging whole-group activities (Li et al., 2014). Another frequent indicator of quality in HICs is student participation, including asking questions of the teacher, which aligns with the value western cultures place on student curiosity and individual initiative. But in cultures that value the authority of teachers, student attentiveness and ability to memorize may be more prized.

Learning environments are shaped by cultural values and the demographics and economy of a society. For example, in China, teachers agree that a high-quality ECE classrooms should be orderly and “quiet” (Hu et al., 2016). Teachers value strict classroom management because of large class sizes and the need to ensure that all children can hear. This view is not reflected in mainstream ECE assessment tools in the United States, and few existing assessment tools accurately portray meaningful pedagogical interactions in such a context. If some high-quality indicators are systematically unobserved in classrooms in LMICs, are these measurements fair and reliable in these contexts? Are low scores indicative of low quality, or are they indicative of irrelevance? While there is utility in monitoring ECE systems comparably across countries, the notion of using the same metrics to compare quality across countries and contexts may itself be driven by a Western perspective.

### Empirically Rigorous

Empirical research is crucial for understanding the utility of a measure. Key criteria include whether measures reflect authentic domains of quality (as reflected partially by psychometric analyses), are sufficiently sensitive, and predict children’s development. How sensitive a measure is to changes in quality, and how strongly it explains children’s outcomes, are important criteria for guiding policy and teacher training. For research purposes, there is also a requirement that a tool can be reliably implemented and is adequately sensitive to detecting variation in teaching practice. Within LMICs, it is important that items reflect variation in the population of interest (e.g., removing items with floor and ceiling effects). Finally, whether the results of standardized measures are cross-culturally comparable is another vital topic in need of empirical investigation. In using tools across different countries and cultures, some revisions are likely to be necessary. How such adaptations affect the cross-cultural comparability of a tool is important to examine.

## Measuring ECE Quality in LMICs

ECE quality in LMICs has been primarily studied using three widely applied observational assessments: Early Childhood Education Rating System (ECERS), Classroom Assessment Scoring System (CLASS), and Measuring Early Learning Environments (MELE). We also discuss a fourth tool, the Teacher Instructional Practices and Processes System (TIPPS), given the unique longitudinal studies conducted with it. These tools fall into two categories: developed in HICs and adapted for use in LMICS (ECERS and CLASS), and developed specifically for LMIC settings (MELE and TIPPS). MELE and TIPPS were developed with the aim of being universal tools for LMIC contexts with room for adaptation as needed. We consider the theoretical grounding, contextual-adaptation, and empirical rigor in the studies we review.

### Adapting Existing Tools

#### ECERS-R (ECERS-3)

ECERS and its revised versions have been used in at least 20 countries, including Columbia, Chile, China, India, Kenya, Zanzibar, and Uganda. A notable feature is the tools’ grounding in education theories (e.g., self-determination theory and attachment theory) developed in industrialized countries (Clifford et al., 2010) that are untested in LMICs. For example, ECERS-R gives particular value to individualized learning and free play (Harms et al., 2015), which are rarely seen in LMICs, in part due to large class sizes and a paucity of teachers (Li et al., 2014). The ECERS-R includes seven subscales (space and furnishings, personal care routines, language-reasoning, activities, interaction, program structure, and parents and staff) and has been modified in a few developing countries. For example, researchers added items related to structural quality in poor areas (e.g., Bangladesh; Aboud, 2006). Researchers in China and Cambodia modified ECERS-R for their contexts to emphasize collective activities and whole-group teaching (Rao and Pearson, 2007; Li et al., 2014).

Importantly, studies in HICs have not verified the factor structure of several or all components of ECERS-R through rigorous psychometric analysis (e.g., Gordon et al., 2013; Mayer and Beckh, 2016). We highlight two studies that examined the factor structure in LMICs (both upper-middle-income countries). In Colombia, Betancur et al. (2021) could not replicate the seven-factor structure using principal components analysis, and instead identified three new factors: materials and space, interactions, and routines and practices. In Brazil, Mariano et al. (2019) also found that a similar three-factor model fit the data best. Although the existing research evidence from LMICs is insufficient to draw definitive conclusions, the findings suggest that the structure of the original ECERS-R could not be verified in two developing countries.

Regarding associations with child outcomes, the studies that exist are limited to cross-sectional studies. In China (Li et al., 2014) and Cambodia (Rao and Pearson, 2007), adapted versions of ECERS-R positively predicted children’s emotional, language, mathematics, creative, and motor skills (*r*=0.2–0.3SD). In Colombia, Betancur et al. (2021) found both positive and negative correlations between three identified quality dimensions and child outcomes (*r*=−0.14–0.31).

More research is needed to understand the different dimensions of classroom quality that manifest using the ECERS-R across diverse settings. In addition, rigorous, longitudinal evidence is needed to examine if domains of process quality as measured by the ECERS-R are related to child outcomes in LMICs beyond correlations.

#### CLASS

The CLASS is one of the most widely researched classroom observation tools, and empirical studies in the United States have validated the organization of teacher-student interactions into three major domains: Emotional Support, Classroom Organization, and Instructional Support (Hamre et al., 2014). Outside of a HIC context, published studies on the CLASS have primarily been in Latin America (all in currently high- or upper-middle-income countries) and China (an upper-middle-income country). Jensen et al. (2020) used mixed methods to examine the ecological validity of the CLASS factor structure in 58 pre-primary classrooms in Central Mexico. They found that an alternative three-factor model fit the data best (Emotional Support, Social Relationships for Teaching, and Instructional Interactions) and suggest that a unified approach to validity is needed to develop, adapt, and refine measures to new contexts. In China, in a sample of 180 kindergarten classrooms, Hu et al. (2016) concluded that the original three-factor structure had acceptable psychometric properties and was appropriate for use. However, a second study in mixed-age ECE settings in poverty-stricken areas of China found difficulties in using the CLASS, including severe floor effects in the Emotional Support and Instructional Support domains (Wang, 2009), indicating heterogeneity within countries as well. Finally, in Kosovo and Ukraine (two LMICs in Europe), Von Suchodoletz et al. (2020) found acceptable fit with the CLASS 3-factor model after dropping the Negative Climate dimension, with significant variation within each country and low levels of quality overall. They found no associations between structural quality characteristics and process quality.

In both Chile and Ecuador, researchers concluded that the CLASS was psychometrically similar in factor structure to the originally proposed model and predicted some child outcomes in large samples of pre-primary and kindergarten classrooms using longitudinal data, but with small associations between classroom quality and child outcomes (*d*=0.07–0.11; Leyva et al., 2015; Araujo et al., 2016). Araujo et al.’ (2016) study was unique for its rigor; researchers randomized 24,000 Ecuadorian children to kindergarten classrooms and examined how classroom quality—measured as Responsive Teaching by the CLASS—causally impacted child outcomes.

Research on the CLASS across a diverse set of LMICs is sparse, particularly in low-income countries. But the studies to date suggest it can measure underlying domains of quality as well as predict child outcomes in LMICs. Given that the CLASS has not yet been used successfully in Asia or Africa, research on the ECERS tools may be more fruitful.

### Creating New Tools

#### MELE

MELE was explicitly designed for use in LMICs and includes the expectation that researchers from different countries will adapt the tool. MELE outlines “core” domains of quality learning environments and provides examples of items in existing tools for each domain (UNESCO et al., 2017). MELE includes seven domains: play, pedagogy, interactions, environment, parent/community engagement, personnel, and inclusion (UNESCO et al., 2017). Because the structure and items are flexible, the versions used by researchers in various countries differ. For example, four domains were used in rural China (Su et al., 2021). Three different domains were used in a cross-country study in sub-Saharan Africa (Raikes et al., 2020), while seven domains were used in Tanzania (Anderson and Sayre, 2016). These studies all report that MELE has acceptable psychometric properties, though rigorous psychometric analyses have been sparse.

Three cross-sectional studies have examined links with child outcomes. Su et al. (2021) used a four-subscale version of MELE in China and found small associations between three of the subscales and child outcomes (*d*=0.09–0.10). They also found stronger correlations in rural versus urban areas. In Tanzania, Raikes et al. (2020) found that only one of the three of seven domains examined had a small correlation with child outcomes (*f*^2^=0.02; Raikes et al., 2020). Finally, Betancur et al. (2021) employed a revision of MELE in Colombia that added new domains and items to capture opportunities to engage in art, play, literature, and exploration, in alignment with national ECE pedagogical goals. Using a nationally representative sample, they found pedagogical quality, language activities, and early math activities positively predicted child outcomes (*d*=0.05–0.16).

More psychometric studies are needed to examine how the domains as suggested by MELE manifest across countries, but the adaptation of the tool within each study makes this a challenging task. In addition, longitudinal studies that can move beyond correlations are needed to examine if classroom quality as measured by the MELE predicts child outcomes.

#### TIPPS

TIPPS was designed for use in LMICs (Seidman et al., 2013) and has 19 items focused exclusively on teacher-child interaction quality. Only two studies on the TIPPS-ECE have been published, both in Ghana. The first found that a three-factor model covering Facilitating Deeper Learning, Emotional Support and Behavior Management, and Supporting Student Expression fit the data well (Wolf et al., 2018). This study, as well as a second, used longitudinal data to test the predictive power of these three factors for early childhood learning and development over one school year, and both found evidence of small associations (*d*=0.05–0.10; McCoy and Wolf, 2018; Wolf et al., 2018). The tool holds promise as one that can capture meaningful variation in learning environments in LMICs, but more research is needed to examine the factor structure across diverse settings, its associations with child outcomes, and its applicability across different LMICs.

## Discussion

We review the available research on measuring ECE process quality in LMICs. The current set of widely used tools may miss important elements, and (if allowed) leave adaptation in the hands of each user. There is a small but growing evidence base on the utility of “bottom-up” classroom observation tools to capture important elements of classroom quality, but these also raise challenges of comparability across contexts as there has yet to be systematic documentation of adaptation processes (see Ponguta et al., 2019, for an exception). Such information could generate knowledge about how to capture elements of the classroom context that are differentially valued across diverse settings. We suggest that a new wave of research is needed that incorporates theoretical grounding, local adaptability, and empirical rigor, as well as careful documentation of the types of adaptations implemented when that is the case. Without such efforts, understanding true variation in early learning opportunities for children around the world—as well as inequalities within countries—is not possible.

This work will need to balance the tension between western and local views of what constitutes “high-quality,” and consider how local realities shape teacher-child interactions. In HIC contexts, for example, student-centered and play-based learning are often emphasized (Hirsh-Pasek et al., 2009). These elements of quality as measured through direct observation are infrequent in LMICs (e.g., Araujo et al., 2016). And in some LMIC contexts, such as China, teacher authority and student silence are highly valued. While documenting the lack of opportunities for certain types of learning is important, equally so is capturing variation in existing practices.

The few studies that have examined associations between observed classroom quality and child development in LMICs have used cross-sectional data; rigorous longitudinal studies are needed. Accumulated knowledge from US-based literature shows that while correlational studies show associations between classroom quality and child outcomes, high-quality studies and meta-analyses show weak—or even null—associations (Burchinal, 2018). Given this, what are the expectations for the predictive validity of classroom quality on child outcomes in LMICs? Recent research finds the home environment, rather than country environment, is the most important input to early childhood human capital formation (Schoellman, 2016). At the same time, it is possible that with lower levels of home stimulation, particularly in south Asia and sub-Saharan Africa (McCoy et al., 2018), rigorous studies may show large effects. In addition, critical dimensions of process quality in LMICs may not yet be identified, further limiting the predictive power of current classroom observation tools.

As ECE continues to expand within many countries, there have been minimal efforts to develop national monitoring systems that focus on process quality (Yoshikawa et al., 2018). Policymakers need fast-response research with high-quality empirical evidence to guide the improvement of ECE systems (Weiland et al., 2021). To avoid repeating the cycle of expanding access and only later attending to quality, a global research agenda focused on ECE quality is needed. “Leapfrogging” in education entails harnessing innovation and current knowledge accumulation to accelerate progress (Winthrop et al., 2018). As it applies to measuring ECE quality, this can include building on existing knowledge to focus monitoring systems on process quality, examine quality thresholds as they relate to child outcomes (Hatfield et al., 2016), and examine whether and how structural quality enables process quality (Vandell and Wolfe, 2000) in low-resource settings. If items capturing the desired constructs cannot be found, it may also be possible to develop new, contextually specific items to add to a “core” set of items by working with local stakeholders, experts in child development, and psychometricians.

Following suit from the Measuring Early Learning and Quality Outcomes initiative (UNESCO et al., 2017), we suggest a core set of items to be used globally, with a flexible module to be added that can be adapted to meet local needs and realities. Empirical studies will be needed to understand the utility of these modules in producing reliable and valid measures of distinct dimensions of the classroom context. Such efforts are needed to ensure that the expansion of ECE globally meets its potential to improve the development and learning of all children.

## Author Contributions

SC and SW equally contributed to the design and implementation of the study, analysis of the results, and writing of the manuscript. All authors contributed to the article and approved the submitted version.

## Funding

The writing of the manuscript was funded by the NAEd/Spencer Postdoctoral Fellowship to SC and SW. The publication of the manuscript was funded by China New Higher Education Group to SC.

## Conflict of Interest

The authors declare that the research was conducted in the absence of any commercial or financial relationships that could be construed as a potential conflict of interest.

## Publisher’s Note

All claims expressed in this article are solely those of the authors and do not necessarily represent those of their affiliated organizations, or those of the publisher, the editors and the reviewers. Any product that may be evaluated in this article, or claim that may be made by its manufacturer, is not guaranteed or endorsed by the publisher.
